# Natural course of talar avascular necrosis during short-term follow-up and factors associated with Disease progression

**DOI:** 10.1186/s12891-023-07136-9

**Published:** 2024-01-22

**Authors:** Yoon Hyo Choi, Tae Hun Kwon, Ji Hye Choi, Dong Yeon Lee, Kyoung Min Lee

**Affiliations:** 1 Department of Orthopedic Surgery, Seoul Nationl University Bundang Hospital, 300 Gumi-Dong, Bundang-Gu, Seongnam-Si, Gyeonggi South Korea; 2 Department of Orthopedic Surgery, Korea University Anam Hospital, Seoul, South Korea; 3 Department of Orthopedic Surgery, Seoul National University Hospital, Seoul, South Korea

**Keywords:** Avascular necrosis, Talus, Natural history, Progression, Radiographic

## Abstract

**Background:**

This retrospective cohort study aimed to investigate the natural history of talar avascular necrosis (AVN) during short-term outpatient follow-up and to identify the risk factors for progression to collapse and arthritic changes.

**Methods:**

Thirty-four cases of talar AVN from 34 patients (15 males, 19 females) were included. The mean age of the patients was 48.9 years (SD 16.0 years) and the mean follow-up period was 39.5 months (SD 42.0 months). The patients were divided into two groups i.e., progression and non-progression groups. The progression group consisted of those who showed aggravation of the Ficat stage during the follow-up period or advanced arthritis of the ankle joint (Ficat stage 4) at presentation. Demographic data and information regarding BMI, medical comorbidities, trauma history, bilaterality, and location of the lesion (shoulder vs. non-shoulder lesions) were collected. Following the univariate analysis, a binary logistic regression analysis was performed.

**Results:**

The location of the talar AVN was the only significant factor (p = 0.047) associated with disease progression. A total of 14.3% (2 of 14) of the central (non-shoulder) talar AVN lesions showed progression, while 50% (10 of 20) of shoulder lesions aggravated during follow-up. Age, sex, bilaterality, medical comorbidities, and trauma history were not associated with progressive talar collapse or subsequent arthritic changes in talar AVN.

**Conclusions:**

Conservative treatment should be considered for a central lesion of the talar AVN because it tends to remain stable without progression. A more comprehensive study with a larger study population is required to establish the surgical indications for talar AVN.

**Level of evidence:**

Prognostic level III.

## Introduction

Avascular necrosis (AVN) of the talus is rare, and its true prevalence is unknown; it is assumed to account for about 2% of all symptomatic cases of AVN [1, 2]. However, the disease frequently leads to a debilitating condition characterized by severe ankle pain and loss of range of motion, negatively impacting gait and overall function [3]. Additionally, it is associated with structural collapse and arthritic changes, ultimately necessitating joint-sacrificing strategies [1]. Known etiologies of the disease include trauma and medical comorbidities, of which 75% comprise traumatic causes, such as talar neck fracture. Hawkins et al. reported that the incidence of talar AVN was 53% in 57 talar neck fractures [4]. Medical comorbidities, such as alcoholism, corticosteroid use in systemic lupus erythematosus, organ transplant states, and uricemia, impede blood flow to certain parts of the body, making patients vulnerable to talar AVN [5–7]. Meanwhile, iatrogenic talus AVN is rarely reported [8, 9].

The progression of talar AVN is represented by the Ficat classification [10–12]. For advanced stages with collapsed or arthritic ankles, joint sacrificing treatment, such as arthrodesis or joint replacement, may be done, while revascularization procedures such as multiple drilling or vascularized bone graft could be considered in the early stage of talar AVN without collapse. Recently, talar prostheses have shown promising outcomes and functional scores [13–15]. However, further large-scale research is needed in this regard [11]. Although previous studies have reported satisfactory results for each surgical procedure, it remains debatable whether the revascularization procedure could effectively restore circulation to the bone.

Furthermore, the natural course of this disease is not well established because of its rare incidence This general lack of knowledge regarding the factors that influence disease progression makes it challenging to develop effective management strategies. If the disease is misdiagnosed or overlooked, patients with certain predispositions are at a heightened risk of rapid progression of the condition. This knowledge gap also hinders the evaluation of the clinical utility and cost-effectiveness of specific surgical procedures, particularly in the early stages of talar AVN. The difficulty in weighing the risks and benefits, especially of revascularization procedures with uncertain outcomes, further complicates surgical decision-making. Understanding the risk factors for disease progression or poor prognosis is crucial for surgeons to tailor management strategies and set appropriate surgical indications. This insight is also vital for analyzing literature reporting surgical outcomes of talar AVN [11]. Previous studies have suggested that the location and size of talar AVN are closely related to disease progression, but this has not been definitively confirmed [1].

Therefore, the objective of this study was to elucidate the risk factors, encompassing both patient characteristics and the location of talar AVN, that contribute to collapse and progression during follow-up in patients who underwent conservative management.

## Materials and methods

### Patients

We retrieved and reviewed the data of patients with talar AVN who visited our orthopedic department between January 2004 and December 2021. We collected the demographic data of the patients and information regarding BMI, alcohol consumption, history of corticosteroid use and recurrent sprain, comorbidities, trauma, and presence of AVN of other joints. Patients with talar AVN underwent weight-bearing ankle anteroposterior (AP), lateral, and mortis X-rays. For cases with uncertain diagnosis after X-ray or CT imaging, magnetic resonance image (MRI) examination was additionally performed. Patients with post-infectious talar AVN or non-ambulatory patients were excluded from the study. For patients with bilateral lesions, only the side with the more severe talar lesion or greater pain, or alternatively the dominant limb, was selected for inclusion in the data analysis.

All patients underwent conservative management and periodic radiographic examinations. Conservative management included pain control, tolerable weight-bearing ambulation with or without crutches, and short-term application of a short leg removable splint when the patients’ symptoms were aggravated.

### Classification and definition of terms

**Fig. 1:**
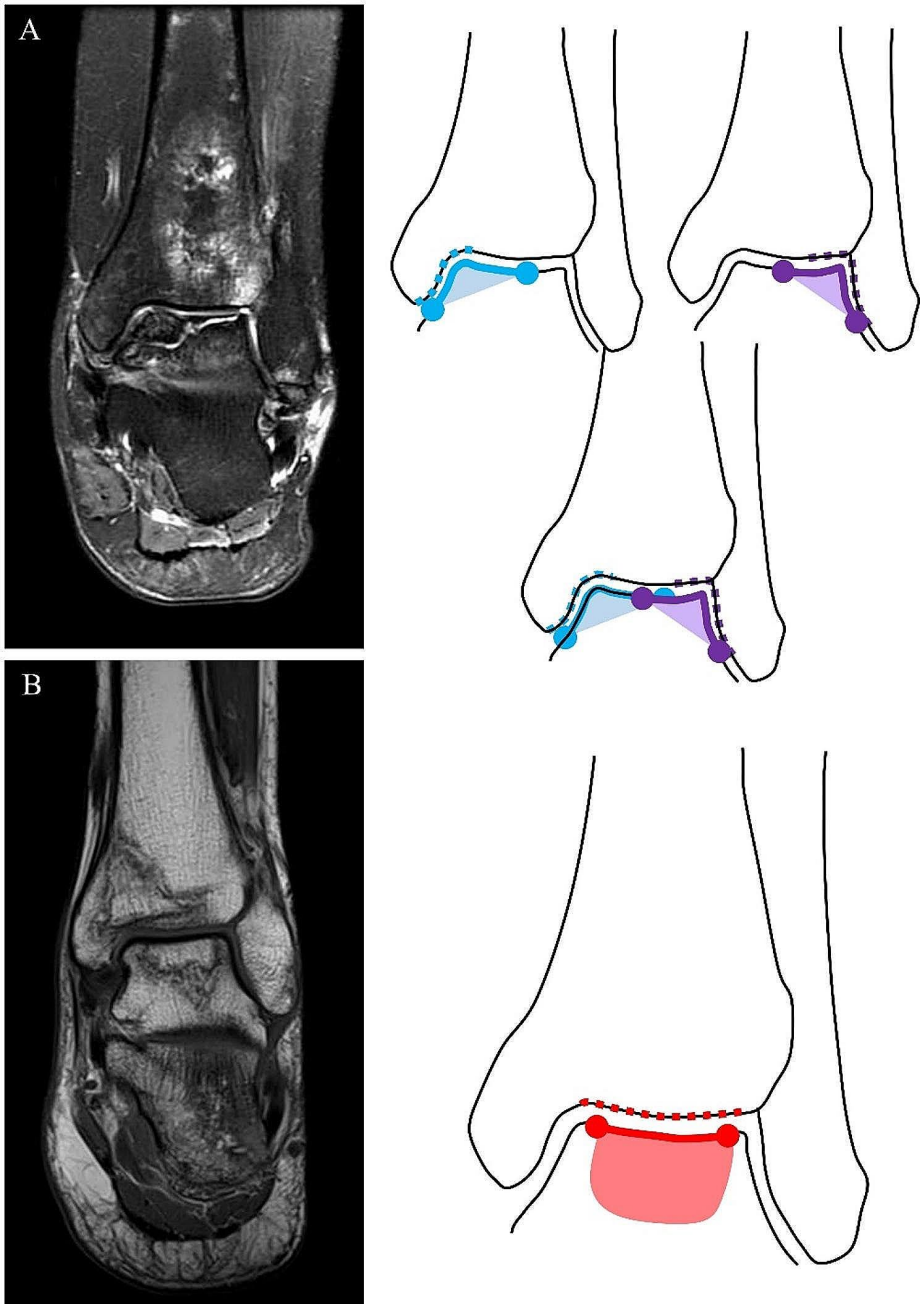
**A**: Shoulder lesion of talar AVN involves either the medial or lateral talar shoulder. **B**: Non-shoulder lesion of talar AVN involves the central part of talar dome without shoulder area involvement

We defined the progression group as patients showing aggravation of the Ficat stage [10, 16] during the follow-up period or advanced arthritis of the ankle joint (Ficat stage 4) at presentation. The non-progression group constituted of those with lesions at the same stage as that during follow-up. The locations of talar AVN were categorized as either ‘talar shoulder’ or ‘central talar dome,’ based on ankle AP X-ray or coronal images from CT or MRI. Central talar dome lesions were identified when osteonecrotic lesions appeared centrally in the talar dome without involving the talar shoulders. In other words, the extent of the AVN did not surpass the tibial plafond and remained within the tibiotalar articulation. Conversely, ‘talar shoulder lesions’ were defined as AVN involving either the medial or lateral talar shoulder, or, put differently, eccentrically affecting one or both gutters and extending to the talar dome (Fig. 1). The classification of each lesion was independently determined by two individuals following these criteria. The inter-observer reliability demonstrated excellent results, with a Cohen’s Kappa statistic of 0.833, which falls into the ‘almost perfect’ category according to Landis and Koch’s benchmarks [17]. If there were ambiguous cases, the senior author reviewed and made the final determination.

### Statistical analysis

Descriptive statistical analyses, including the average and standard deviation, were performed. Data normality was evaluated using the Kolmogorov-Smirnov test. Categorical data were analyzed using the chi-squared test or Fisher’s exact test. Continuous variables were compared between the two groups using Student’s t-test or Mann-Whitney U test. For the multivariable logistic regression analysis, a two-step approach was employed to identify significant risk factors associated with the progression of talar AVN. This approach began with a univariate analysis, selecting candidate independent variables that had a p-value of less than 0.1. All statistical analyses were performed using SPSS (version 20.0; IBM Corp., Armonk, NY, USA), and a p-value < 0.05 was considered significant.

## Results

For this study, 34 cases of talar AVN in 34 patients were included in the analysis. Twenty-two patients were included in the progression group, and 12 in the non-progression group. The mean age of the patients was 48.9 years (SD 16.0 years) and there were 15 male and 19 female patients. The mean follow-up period was 39.5 months (SD 42.0 months). Nine patients had bilateral lesions and 25 had unilateral lesions. Eleven patients had traumatic causes and 23 had medical comorbidities. There were 5 cases at Ficat stage 1, 14 cases at Ficat 2, and 10 cases at Ficat 3 and 5 cases at Ficat 4 at the initial presentation (Table 1).

Twenty-two patients belonged to the progression group, whereas 12 did not. The age (p = 0.788) and follow-up period (p = 0.106) were not significantly different between the progression and non-progression groups. Sex (p = 0.097) and location of the lesion (p = 0.066) were the candidate variables associated with progression of talar AVN (Table 1).

**Table 1:** Data summary and comparison between progression and non-progression groups

	Non-progression (N = 22)	Progression (N = 12)	P value
Demographics			
Age	49.5 ± 16.1	47.9 ± 16.6	0.788
Follow-up (months)	30.8 ± 42.3	50.2 ± 38.4	0.106
Sex			0.097
Female	10 (45.5%)	9 (75.0%)	
Male	12 (54.5%)	3 (25.0%)	
BMI (kg/m^2^)	23.6 ± 4.2	25.8 ± 5.7	0.268
Bilateral lesions			0.687
Present	5 (22.7%)	4 (33.3%)	
**Etiology**			
Alcoholics	3 (13.6%)	1 (8.3%)	1.000
Steroid Use	4 (18.2%)	4 (33.3%)	0.410
Post-traumatic	6 (27.3%)	4 (33.3%)	0.714
Recurrent sprain	5 (22.7%)	1 (8.3%)	0.389
SLE	2 (9.1%)	4 (33.3%)	0.154
**AVN characteristics**			
Location			0.066
Non-shoulder lesion	12 (54.5%)	2 (16.7%)	
Shoulder lesion	10 (45.5%)	10 (83.3%)	
Involvement of other joints			1.000
Present	5 (22.7%)	3 (25.0%)	

AVN, avascular necrosis; SLE, Systemic Lupus Erythematosus

In multivariable logistic regression analysis, the location of the lesion was found to be the only significant factor associated with the progression of joint destruction in talar AVN (p = 0.047) (Table 2).

**Table 2:** Factors associated with the progression of talar AVN in binary logistic regression

Variable	Odds ratio	95% CI	P value
Location of AVN (Shoulder)	6.00	1.06, 34.00	0.047
Sex (Male)	0.28	0.06, 1.31	0.107

## Discussion

This retrospective study investigated factors associated with progressive collapse and articular destruction in talar AVN during follow-up of patients who underwent conservative management. Involvement of the talar shoulder was found to be a significant risk factor for the progression of talar collapse and subsequent arthritic changes in the ankle joint. Other factors, including medical comorbidities, were not associated with talar AVN progression.

Although talar AVN is a rare condition, it has drawn the attention of surgeons, owing to the complex nature of the disease and uncertain treatment outcomes. The unknown natural history and aggravating factors have contributed to the difficulty in interpreting the efficacy of a specific treatment. Furthermore, arthritic changes from the talar AVN are usually not subject to motion-preserving surgery, such as total ankle replacement arthroplasty due to collapsed poor bone stock, rather to motion-sacrificing surgery, such as ankle arthrodesis [1, 18]. This effect is considered detrimental to the subsequent quality of life [14].

**Fig. 2:**
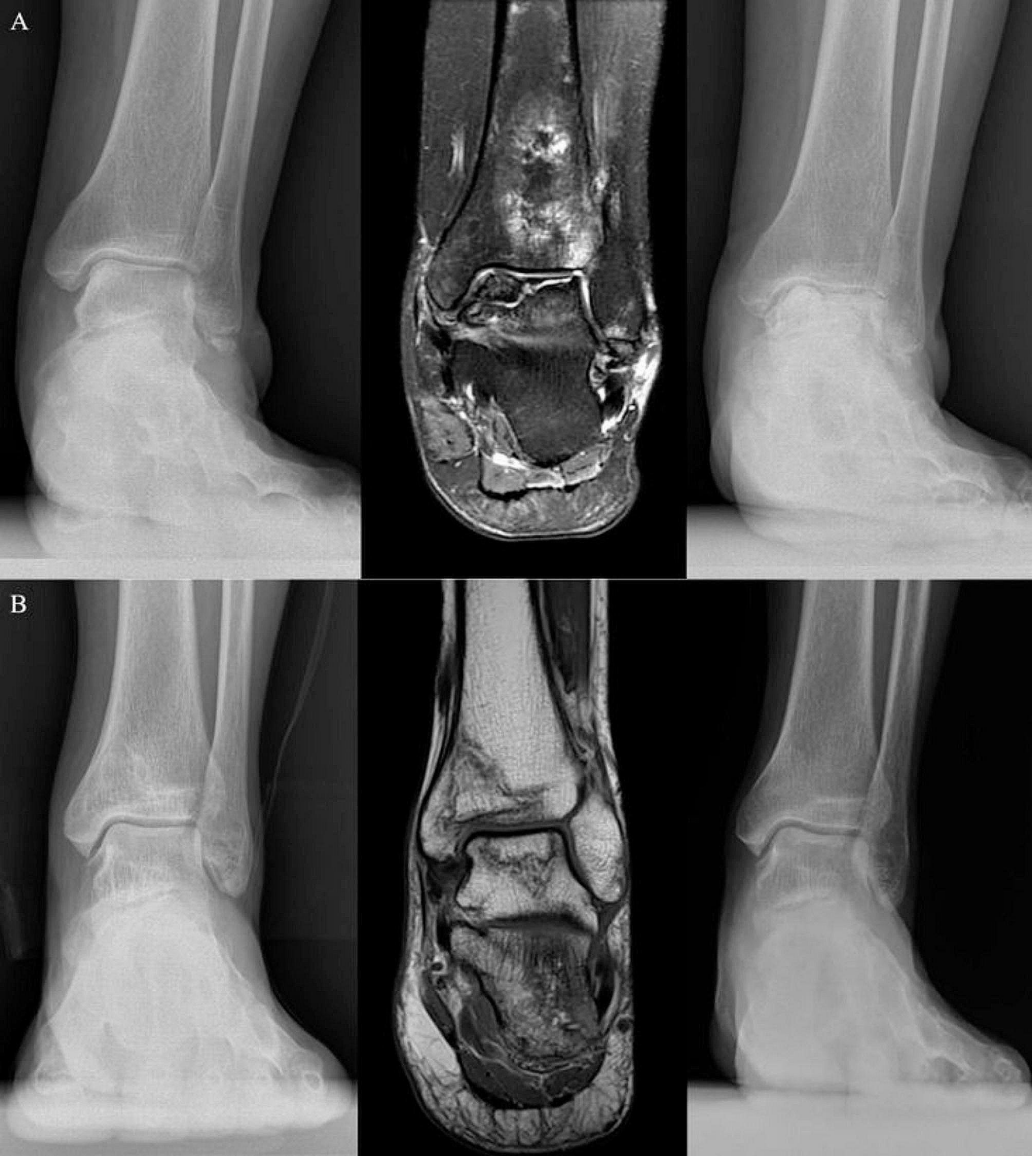
**A**: A shoulder lesion of talar AVN in a 65-year-old female shows structural collapse during 4 months of follow-up. **B**: A non-shoulder lesion of talar AVN in a 54-year-old male remained stable without collapse or arthritic change during 44 months of follow-up

A prior study suggested that the ultimate cause of AVN is a disruption in the bone’s blood supply. The initiating factor, however, can significantly differ, being influenced by factors such as the patient’s genetic predisposition, associated risk factors, and pre-existing medical conditions [1, 3, 19]. In our research, we found that the specific location of the osteonecrotic lesion in the talus was the sole significant risk factor for the advancement of talar AVN (Fig. 2). Contrary to earlier research, established causative factors of talar AVN, such as trauma and medical comorbidities, did not correlate with the progression to structural collapse and subsequent arthritic changes. Notably, our cohort was composed of 29.4% traumatic and 70.6% atraumatic cases. This differs from the usual prevalence of talar AVN, where traumatic causes like talar neck fractures account for about 75% of cases [11]. This unique composition of our cohort should be taken into account when evaluating our study’s findings.

Around 60% of the talar surfaces are known to be covered by articular cartilage, with scanty soft tissue attachments making this bone prone to ischemic insult [20]. The medial one-third of the talar dome is mainly supplied by the branches of the posterior tibial artery, and the lateral one-third is mainly supplied by the branches of the tarsal sinus artery, which are considered to supply shoulder lesions of the talus. The middle third is supplied by four different arteries, including the dorsalis pedis artery, posterior tubercle, tarsal sinus, and tarsal canal branches, which are involved in non-shoulder central lesions [21]. A recent cadaveric study of subchondral vascularity of the talar dome showed that the blood supply to the central talar portion is superior to the medial and lateral talar domes [22], which could explain why shoulder lesions are more prevalent (20 shoulder lesions out of all 34 cases) as compared to non-shoulder lesions in our study.

A previous study reported that the ankle joint contact area is located over the lateral and medial margins of the talus rather than the central region of the talar dome, which corroborates with our results showing that shoulder lesions are more prone to structural collapse and subsequent arthritic [23]. However, another study showed that the contact area was dependent on the ankle position, where the broad contact area in dorsiflexion was distributed over the medial and lateral talar shoulders, and a narrower contact area in plantar flexion was located more centrally [24]. In addition, the contact area was more medially located in supination and more laterally located in pronation [24]. Considering that several experimental studies of ankle joint contact characteristics reported various results depending on the different methodologies and loading conditions, this issue needs further investigation. Future studies should focus on the patients’ daily activity and loading patterns of the ankle joint.

Previous studies reported that protection of talus from weightbearing in precollapse stage of AVN for a long period of time over 9 months improved clinical outcomes [4, 25]. In our study, the patients were allowed to bear weight as per the tolerability, along with pain medication as required. The bilateral lesions did not show significantly worse disease progression than unilateral lesions, although restriction of weight-bearing might have been more difficult in the bilateral lesions. These results might be contradictory to those of previous studies; however, the chronologic stage of the disease and bone strength need to be considered, which were not evaluated in our study.

There is no consensus on the indications, timing, and methods of surgical treatment for talar AVN, owing to the complex nature of this disease and its low incidence. Understanding the natural history of a disease may prevent unnecessary invasive intervention and help in establishing the appropriate surgical indication, whereby a specific group of patients could be defined as those who would significantly improve with surgical intervention as compared to those without surgical intervention. Based on our study results, surgical interventions to restore blood supply to the talar bone, such as multiple drilling and vascularized bone grafting, should be done with caution if the AVN is located in the central region of the talus without talar shoulder involvement. Only 14.3% (2 of 14) of the central lesions showed disease progression, while 50% (10 of 20) of the shoulder lesions were aggravated during follow-up in our study. Further studies with a larger number of cases and more comprehensive data collection are needed to refine the management and prognosis of talar AVN.

There are some limitations in this study. First, it was a retrospective study conducted at a single tertiary medical center. Therefore, an unknown bias might have affected the study results. Hence, the results should be generalized with caution. Second, in our study, the identification and classification of osteonecrotic lesions were, in many cases, based on the coronal plane using AP X-rays or CT scans. In instances of low-grade talar AVN, some patients did not undergo MRI. As a result, although the location of lesions (identified as either shoulder or central) might be accurately determined, the comprehensive extent and size of these lesions could potentially be underestimated. Given the potential importance of the size of preradiographic AVN in the progression of the disease [1], more extensive radiographic studies, especially those utilizing MRI, are deemed necessary. Third, our study’s small sample size might introduce vulnerability. Even slight variations in the values we defined and measured this time – such as the criteria for progression or the categories of lesion location – could result in confidence intervals that either include 1.0 or become excessively wide. Despite these limitations, considering the rare prevalence and previous studies on talar AVN, we believe that 34 patients with talar AVN in a single institution is not a small number. Fourth, the exact chronological course of talar AVN could not be evaluated, especially in nontraumatic AVN, where the onset of the disease is unclear. Therefore, we could not recommend the exact period of conservative treatment in this study.

## Conclusion

Shoulder lesions of talar AVN were more prone to disease progression, while central lesions without talar shoulder involvement tended to remain stable without further collapse and arthritic changes. Conservative treatment should be considered primarily for patients with central talar AVN lesions.

## Data Availability

The datasets used and/or analyzed during the study are available from the corresponding author on reasonable request.
